# Identification of Amino Acids Essential for Estrone-3-Sulfate Transport within Transmembrane Domain 2 of Organic Anion Transporting Polypeptide 1B1

**DOI:** 10.1371/journal.pone.0036647

**Published:** 2012-05-04

**Authors:** Nan Li, Weifang Hong, Hong Huang, Hanping Lu, Guangyun Lin, Mei Hong

**Affiliations:** 1 College of Life Science, South China Agricultural University, Guangzhou, China; 2 School of Information, University of South Florida, Tampa, Florida, United States of America; 3 Zhongshan School of Medicine, Zhongshan (Sun Yat-sen) University, Guangzhou, China; University of Cambridge, United Kingdom

## Abstract

As an important structure in membrane proteins, transmembrane domains have been found to be crucial for properly targeting the protein to cell membrane as well as carrying out transport functions in transporters. Computer analysis of OATP sequences revealed transmembrane domain 2 (TM2) is among those transmembrane domains that have high amino acid identities within different family members. In the present study, we identify four amino acids (Asp70, Phe73, Glu74, and Gly76) that are essential for the transport function of OATP1B1, an OATP member that is specifically expressed in the human liver. A substitution of these four amino acids with alanine resulted in significantly reduced transport activity. Further mutagenesis showed the charged property of Asp70 and Glu74 is critical for proper function of the transporter protein. Comparison of the kinetic parameters indicated that Asp70 is likely to interact with the substrate while Glu74 may be involved in stabilizing the binding site through formation of a salt-bridge. The aromatic ring structure of Phe73 seems to play an important role because substitution of Phe73 with tyrosine, another amino acid with a similar structure, led to partially restored transport function. On the other hand, replacement of Gly76 with either alanine or valine could not recover the function of the transporter. Considering the nature of a transmembrane helix, we proposed that Gly76 may be important for maintaining the proper structure of the protein. Interestingly, when subjected to transport function analysis of higher concentration of esteone-3-sulfate (50 µM) that corresponds to the low affinity binding site of OATP1B1, mutants of Phe73, Glu74, and Gly76 all showed a transport function that is comparable to that of the wild-type, suggesting these amino acids may have less impact on the low affinity component of esteone-3-sulfate within OATP1B1, while Asp 70 seems to be involved in the interaction of both sites.

## Introduction

The organic anion transporting polypeptides (OATPs, gene symbol *SLCO*) are a family of transporters that mediate sodium-independent transport of a wide spectrum of structurally independent compounds [1]. Substrates of OATPs are mainly amphipathic organic molecules such as bile salts, hormones and their conjugates, toxins, and different drugs. Besides these charged compounds, they also transport uncharged drugs such as glycosides digoxin [2] and ouabain [3]. Because of their broad substrate specificity, wide tissue distribution and the involvement of drug-drug interaction, OATPs have been extensively recognized as key determinants of absorption, distribution, metabolism, and excretion (ADME) of various drugs, xenobiotics and toxins [4], [5]. The first discovered human member of the OATP family was OATP1A2 that was cloned as an ortholog of rat Oatp1a1 [6]. So far there are 11 members of the human OATP family: OATP1A2, 1B1, 1B3, 1C1, 2A1, 2B1, 3A1, 4A1, 4C1, 5A1, and 6A1 [7]–[9]. Some OATPs are expressed ubiquitously; while others, such as OATP1B1 and OATP1B3, are predominantly found in certain organs or tissues. OATP1B1 is the major OATP located at the basolateral membrane of human hepatocytes and plays an essential role in drug clearance from the body [10]. In recent years, more and more drugs have been shown to be substrates of this OATP family member [11].

Although substrate specificity of transporter proteins are under extensive study, the underlying mechanisms for substrate binding and/or recognition remain largely unknown because the crystal structures of most mammalian drug transporters have not yet been solved [12]. Transmembrane domains (TMs) are essential structural features of membrane proteins and have been proposed to be critically involved in the proper function of other transporters such as organic anion transporters (OATs) [13], [14]. It was also shown that TM 8 and 9 in OATP1B1 are critical for its substrate recognition [12]. Several important amino acids were identified in TM10 of OATP1B1. L545 located in TM10 may be facing the putative substrate translocation pathway and interact directly with substrates. In contrast, F546, L550, and S554 are predicted to face the inside of the protein and most likely affect normal protein structure. However, only simultaneous mutation of these four residues resulted in significantly reduced OATP1B1-mediated transport [15]. A recent study demonstrated that in OATP1B3, K41 in TM1 and R580 in TM11 were critical for bromosulfophthalein (BSP) transport [16]. According to computer-based hydropathy analysis, OATP1B1 as well as other OATP members are predicted to share a very similar transmembrane domain organization with 12 transmembrane domains and a large extracellular loop 5 with conservative cysteine residues [7]. Computer analysis of OATP sequences revealed that several transmembrane domains such as TM 2, 3, 4, and 11 have high amino acid identity [17], which suggests that these domains are conserved in the whole family and may play essential roles in their transport activity. A large number of single nucleotide polymorphism (SNPs) and other sequence variations have been reported in the *SLCO1B1* gene [18], and it was demonstrated that SNPs located within the transmembrane domains often result in functional changes [19], further suggesting that transmembrane domains are key determinants in the proper transport functions of OATPs.

In the present study, we performed alanine-scanning and site-directed mutagenesis for the study of amino acids within putative TM2 of OATP1B1. Four important amino acids (Asp70, Phe73, Glu74, and Gly76) that are critical for low concentration estrone-3-sulfate (E-3-S) uptake (<1 µM) were identified. Further study suggested that the side chain characteristics of these amino acids are important for maintaining proper transport functions. Asp70, Phe73, and Glu74 maybe localized within the pore-facing side of the transport protein and interact with the substrate, while Gly76 may be important for maintaining the proper structure of the protein. On the other hand, there was a partial transport function for higher concentration of E-3-S (50 µM) in mutants F73A, E74A and G76A, suggesting these amino acids may have less impact on the low affinity component of E-3-S within OATP1B1.

## Methods

### Materials

[^3^H]Estrone-3-sulfate (E-3-S) was purchased from PerkinElmer Life Sciences (Waltham, MA). Sulfosuccinimidyl 2- (biotinamido)-ethyl-1, 3-dithiopropionate (NHS-SS-biotin), and streptavidin-agarose beads were purchased from Thermo Scientific (Rockford, IL). All other reagents were purchased from Sigma except otherwise stated.

### Site-directed mutagenesis

Mutant transporters were generated using the QuikChange Lightning Site-Directed Mutagenesis Kit from Agilent (Santa Clara, CA). The pReceiver M07 vector containing the *SCLO1B1* gene and 3-HA tags at the C-terminus was obtained from Genecopoeia (Rockville, MD) and used as the template for the mutagenesis. All mutant sequences were confirmed by full length sequencing (Invitrogen).

### Cell culture and transfection of plasmid constructs into cells

HEK293 cells were purchased from ATCC (Manassas, VA) and grown at 37°C and 5% CO_2_ in Dulbecco's modified Eagle's medium (Invitrogen, Carlsbad, CA) supplemented with 10% fetal bovine serum. Confluent cells in 48-well or 6-well plate were transfected with DNA plasmid using LipofectAMINE 2000 reagent (Invitrogen) following the manufacturer's instructions. Transfected cells were incubated for 48 hrs at 37°C and then used for transport assay and cell surface biotinylation.

### Uptake assay

Cells in a 48-well plate were used for transport measurement. To each well, uptake solution (125 mM NaCl, 4.8 mM KCl, 5.6 mM D-glucose, 1.2 mM KH_2_PO_4_, 25 mM HEPES, 1.2 mM CaCl_2_, and 1.2 mM MgCl_2_, pH7.4, and [^3^H]E-3-S) was added and the uptake was stopped at 10 min by addition of ice-cold phosphate-buffered saline (PBS) solution. The uptake solution was then aspirated off and the well was rapidly washed with ice-cold PBS solution for three times. The cells were then solubilized in 0.2 N NaOH, neutralized in 0.2 N HCl, and the radioactivity of the cell lysate was measured with a liquid scintillation counter Triathler-Hidex (Hidex, Finland). The uptake count was standardized by the amount of protein in each well.

### Cell surface biotinylation and Western blot

Cell surface expression levels of OATP1B1 and its mutants were examined using the membrane-impermeable biotinylation reagent NHS-SS-biotin. Forty-eight hours after transfection, HEK293 cells in 6-well plates were first washed twice with ice-cold PBS (pH 8.0) and then incubated with 1 ml of NHS-SS-biotin (0.5 mg/ml in PBS) in two successive 20-minute incubations on ice with gentle shaking. The biotinylation process was terminated with 100 mM glycine by incubation on ice for 20 min. The cells were then dissolved on ice for 1 h in 400 µl of RIPA buffer (50 mM Tris, 150 mM NaCl, 0.1% SDS, 1% NP-40, protease inhibitors phenylmethylsulfonyl fluoride, 200 µg/ml, leupeptin, 3 µg/ml, pH 7.4). The cell debris was removed by centrifugation at 12,000× *g* for 20 min at 4°C. Supernatants were transferred to new Eppendorf tubes and protein concentration was measure. To equal amount of protein of each sample, 50 µl of streptavidin-agarose beads were added to bind the biotin-labeled cell membrane proteins. The streptavidin-agarose beads bound protein was released in 4×Laemmli buffer and protein was loaded onto a 7.5% SDS-polyacrylamide electrophoresis gel, then transferred electrophoretically to a polyvinylidene difluoride membrane (Millipore, Billerica, MA). OATP1B1 was detected with an anti-HA antibody (Cell Signaling Technology, Danvers, MA).

### Statistical analysis

Data statistical analysis was carried out using Student's *t*-test. Differences between means are regarded as significant if *p*≤0.05.

## Results

### Alanine-scanning of TM2 amino acids

**Figure 1:**
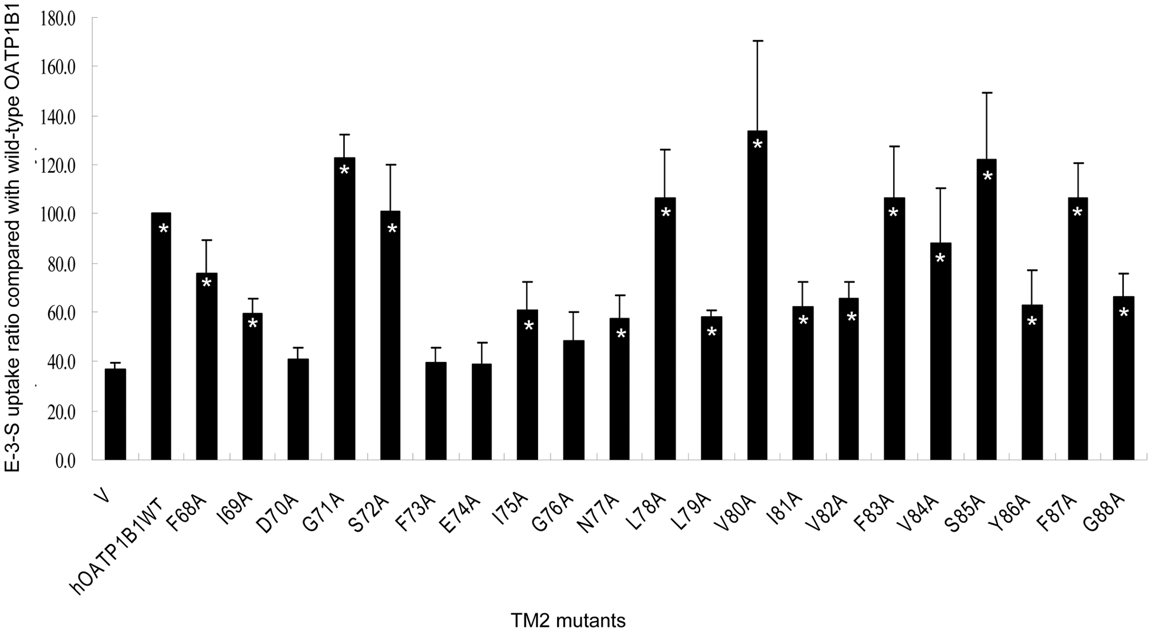
Estrone-3-sulfate uptake of OATP1B1 transmembrane domain 2 mutants. Transport of 100 nM E-3-S in HEK293 cells expressing OATP1B1 and its alanine-substituted mutants was measured. Uptake activity was expressed as a percentage of the uptake measured in wild-type. The results represent data from three experiments, with triplicate measurements for each mutant. The results shown are means ± S.E. (*n* = 3). Asterisks indicate values significantly different (*p*<0.05) from that of mock control (V).

In order to identify the amino acids that are essential for proper transport functions of human OATP1B1, we generated mutants along the predicted TM2 of the protein. The amino acids along TM2 were selected according to Kyte-Doolittle hydrophobicity scale. Therefore, 21 amino acids from position 68 to 88 of OATP1B1 were individually mutated into alanine. The transport activity of the mutants was analyzed using 100 nM of E-3-S as the substrate. As shown in Fig. 1, most of the mutants retained transport function of the substrate and showed significant uptake compared with the mock control (*p*<0.05). However, four mutants, namely Asp70Ala (D70A), Phe73Ala (F73A), Glu74Ala (E74A) and Gly76Ala (G76A), all of which are located on the extracellular half of the transmembrane domain, were found to have significant loss of E-3-S uptake activity. Consequently, further studies were carried out for these four amino acids.

### Protein expression of mutants with reduced transport activity

**Figure 2:**
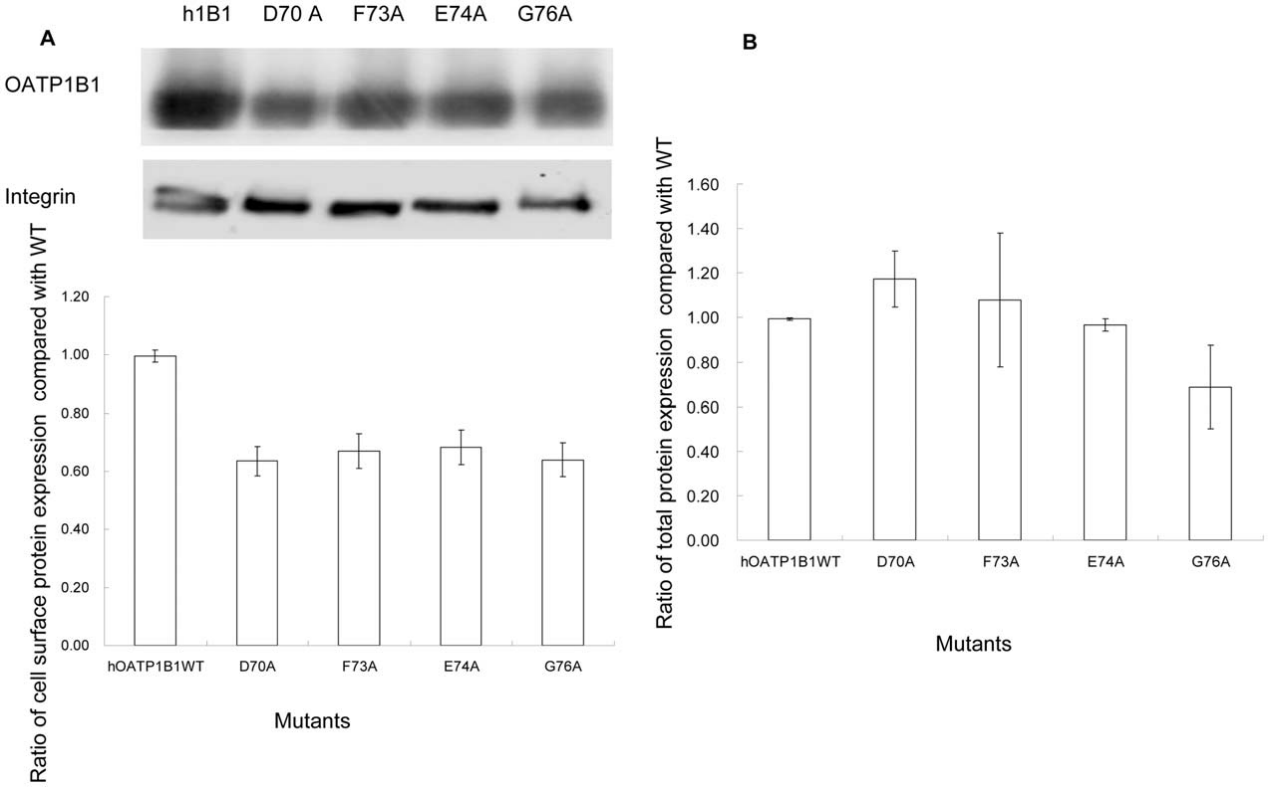
Protein expression of mutants with reduced transport activity. **A.** Cell surface expression of OATP1B1 and mutants. Upper panel, representative blot of OATP1B1 and mutants (D70A, F73A, E74A, G76A). Lower panel, the intensity was quantified relative to the wild-type. Cells were biotinylated, and the biotin-labeled cell surface proteins were precipitated with streptavidin beads, separated by SDS-PAGE, followed by Western blotting with anti-HA antibody. Same blot was probed with integrin antibody as surface protein loading control. **B.** Total protein expression of OATP1B1 and mutants. Cells were lysed with RIPA buffer, separated by SDS-PAGE, followed by Western blotting with anti-HA antibody. The band intensity was quantified relative to the wild-type. The results shown are means ± S.E. (*n* = 3).

Since OATP1B1 is a transporter protein that functions on the cell surface membrane, we first investigated whether the loss of transport function was due to reduced surface expression of the mutant proteins. Cell surface biotinylation was performed using a cell-impermeable reagent NHS-SS-biotin. Our results showed that all four mutants were abundantly (∼60%) expressed on the cell surface (Fig. 2A). We also analyzed total protein expression of these mutants. As indicated in Fig. 2B, D70A, F73A, and E74A had protein expression similar with that of the wild-type, while total protein expression of G76A correlated well with its cell surface expression. This suggested that alternation of Asp70, Phe73, and Glu74 into alanine may have a minor effect on protein expression on the cell surface membrane. However, such a reduction could not account for the significant loss of transport function for these three mutants.

### Effect of mutations of negatively charged Asp70 and Glu74 on hOATP1B1 transport activity

**Figure 3:**
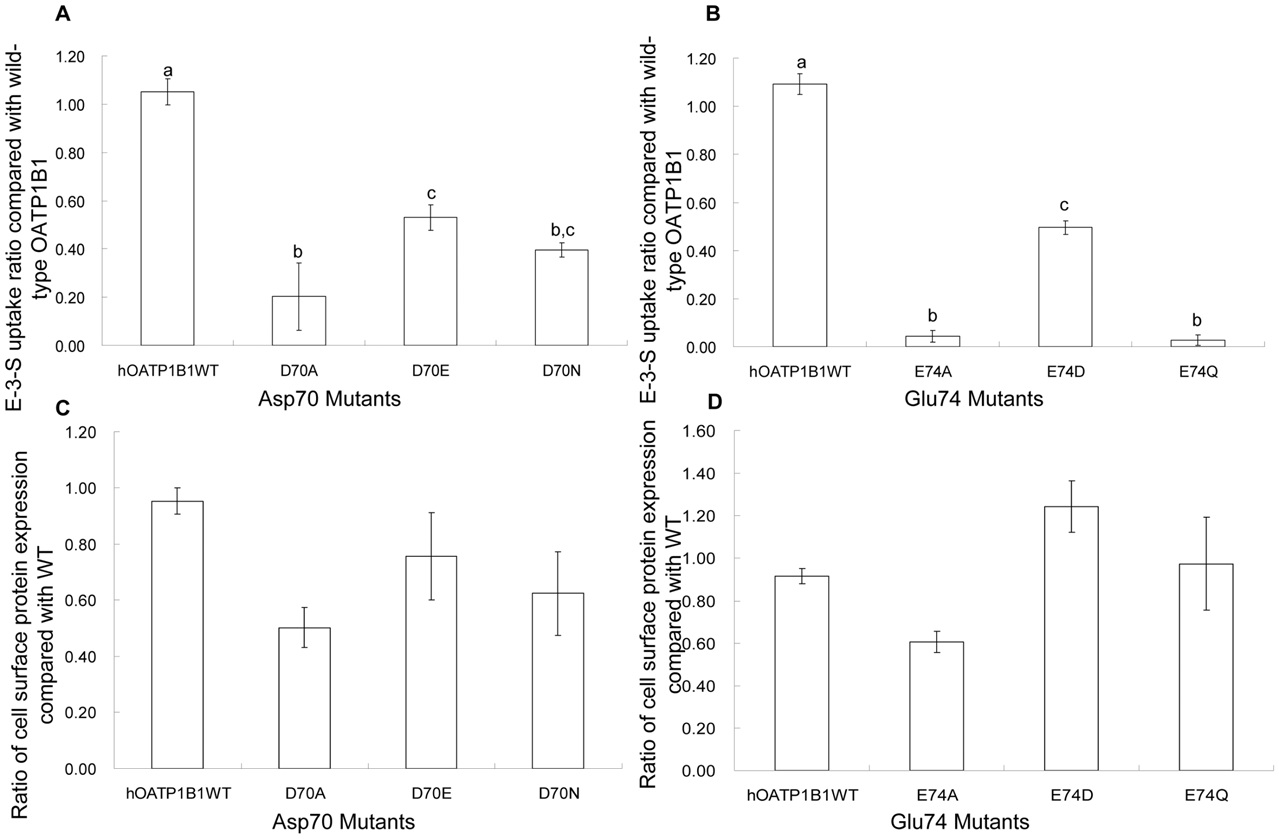
Effects of mutagenesis on negatively charged amino acids. **A.** E-3-S uptake of Asp70 mutants and **B**. Glu74 mutants. Uptake of 100 nM E-3-S was measured in OATP1B1 wild-type and D70A, D70E and D70N as well as in E74A, E74D and E74Q. Net uptake was obtained by subtracting the uptake of cells transfected with empty vector from cells expressing wild-type OATP1B1 or its mutants. Uptake of mutants was normalized to transporter expression at the cell surface in the same batch of cells and quantified relative to that of the wild-type. **C**. Cell surface expression of Asp70 mutants and **D**. Glu74 mutants. The results shown are means ± S.E. (*n* = 3). Different letters indicate values that are significantly different (*p*<0.05).

Among the four critical amino acids identified, Asp70 and Glu74 are two negatively charged amino acids. We then postulated that the charged property of these two amino acids may play a role in proper function of OATP1B1. When Asp70 was mutated into glutamic acid or Glu74 into aspartic acid, there was a significant increase of the uptake activity in comparison with the D70A and E74A mutants. D70E and E74D both showed more than 50% net uptake compared with that of the wild-type OATP1B1 (Fig. 3A, B). We also substituted Asp70 with asparagine, the amino acid with similar structure but non-charged. Although transport activity was elevated, it was to a lesser extent compared with the glutamic acid substitution (Fig. 3A). On the other hand, transport activity of the E74Q mutant was still lacking, similar with that of the E74A substitution (Fig. 3B). The additional mutagenesis of Asp70 and Glu74 resulted in increased protein expression on the plasma membrane (Fig. 3C, D) and the uptake of wild-type OATP1B1 and mutants was therefore adjusted with their cell surface protein expression.

### Effect of mutations of Phe73 and Gly76 on hOATP1B1 function

**Figure 4:**
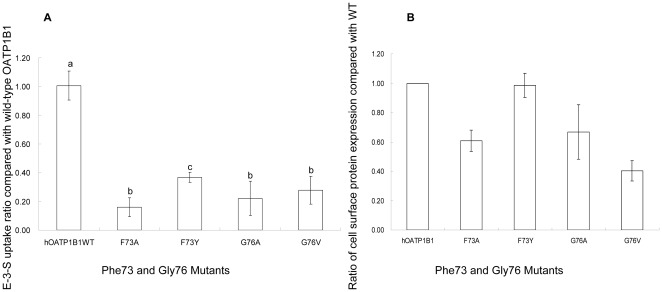
Effects of mutagenesis on Phe73 and Gly76. **A.** E-3-S uptake of Phe73 and Gly76 mutants. Uptake of 100 nM E-3-S was measured in OATP1B1 wild-type and F73A, F73Y as well as in G76A and G76V. Net uptake was obtained by subtracting the uptake of cells transfected with empty vector from cells expressing wild-type OATP1B1 or its mutants. Uptake of mutants was normalized to transporter expression at the cell surface in the same batch of cells and quantified relative to that of the wild-type. **B.** Cell surface expression of Phe73 and Gly76 mutants. The results shown are means ± S.E. (*n* = 3). Different letters indicate values that are significantly different (*p*<0.05).

To further elucidate the molecular mechanism underlying the effect of Phe73 on OATP1B1 transport activity, we mutated this amino acid into tyrosine, another amino acid that contains an aromatic ring and has a similar structure to phenylalanine. Such a mutation resulted in an increase in transport activity that was around 40% of OATP1B1 (Fig. 4A). Cell surface expression of F73Y, on the other hand, was comparable with that of the wild-type (Fig. 4B). Glycine is the amino acid with the smallest side chain. Since the mutation of glycine 76 into alanine, which contains a methyl group as its side group, significantly reduced the transport activity of E-3-S, we further mutated glycine into valine, which has an even larger hydrophobic side chain, to investigate whether the side group size is important for OATP1B1 function. Interestingly, such a substitution did not seem to cause any significant change in the uptake activity (Fig. 4A) or the protein expression on the plasma membrane (Fig. 4B).

### Kinetic analysis of mutants with reduced transport function

**Figure 5:**
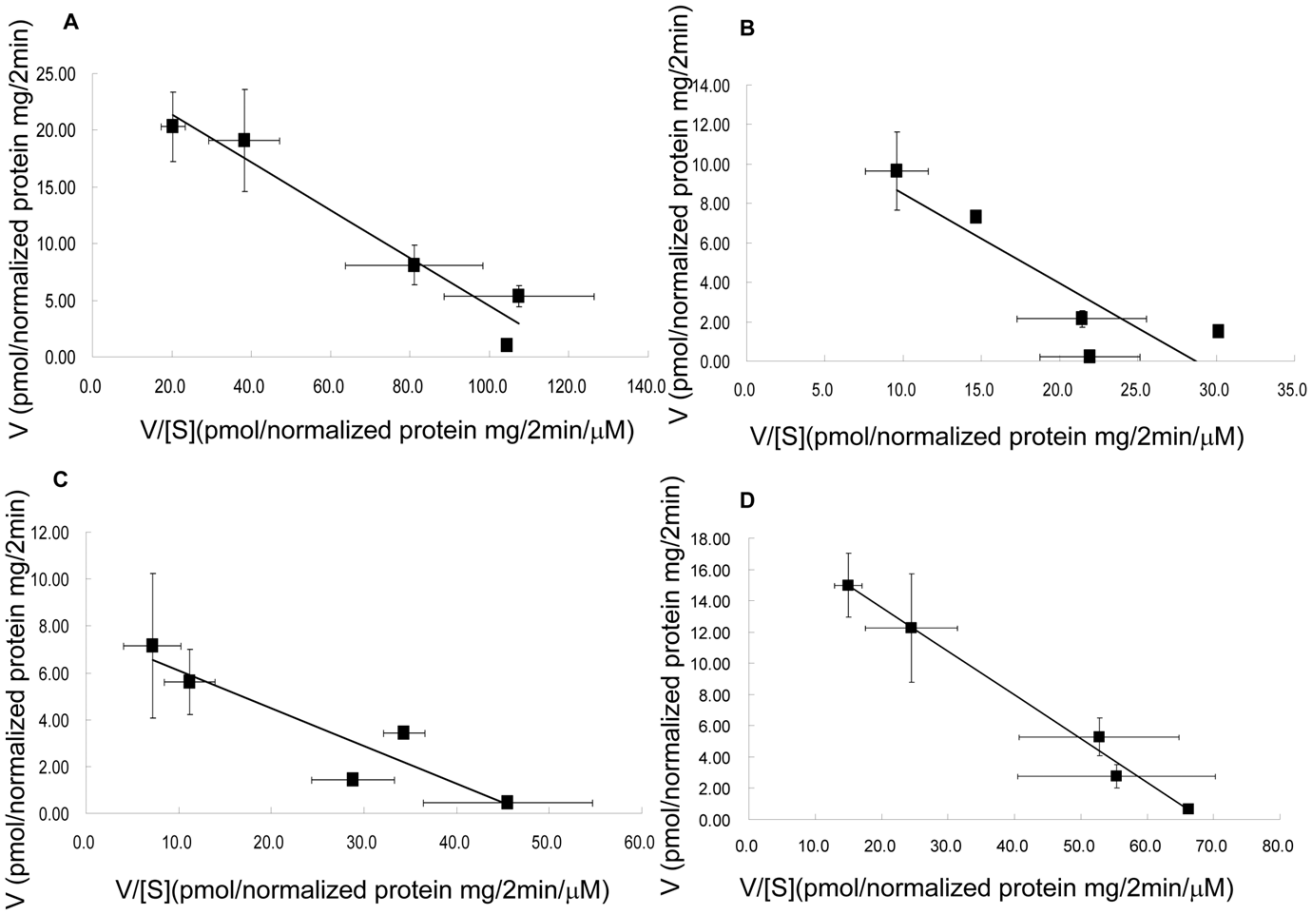
Eadie-Hofstee plots of low concentrations of estrone-3-sulfate uptake by OATP1B1 and its mutants. **A**. Uptake of OATP1B1, **B**.D70N, **C**. F73Y and **D**. E74D. Uptake of E-3-S was measured at concentrations range from 0.01 to 1 µM. Uptake was conducted at 37°C for 2 min with empty vector, OATP1B1 or its mutants in HEK293 cells. Net uptake was obtained by subtracting the uptake of cells transfected with empty vector from cells expressing wild-type OATP1B1 or its mutants. Uptake of mutants was normalized to transporter expression at the cell surface in the same batch of cells and quantified relative to that of the wild-type. The results shown are means ± S.E. (*n* = 3).

Uptake activity of D70A, F73A, E74A, and G76A were all too low for kinetic analysis. Therefore, we analyzed the kinetic properties of D70N, F73Y, and E74D instead (Fig. 5). As shown in Table 1, D70N had a much larger K_m_ (more than twofold increase) and a lower Vmax compared to that of wild-type OATP1B1. On the other hand, E74D had a similar K_m_ as well as V_max_ in comparison with that of the wild-type. The K_m_ for F73Y is slightly lower than the wild-type. Interestingly, though F73Y had a similar cell surface expression as that of wild-type OATP1B1 (Fig. 4B), its V_max_ is only around 30% compared to the wild-type.

**Table 1:** Kinetic parameters of OATP1B1 wild-type and its mutants for low concentrations of E-3-S.

	Km (µM)	Vmax (pmol/mg protein/2 min)
OATP1B1	0.21±0.02	25.6±4.6
D70N	0.46*±0.05	13.1*±3.0
F73Y	0.16±0.11	7.7*±3.3
E74D	0.28±0.03	19.2±4.0

Uptake of E-3-S was measure at concentrations of 10 nM, 50 nM, 100 nM, 500 nM and 1 µM at a 2-min interval for OATP1B1 and its mutants. Transport kinetic values were calculated using the Eadie-Hofstee transformation. The results shown are means ± S.E. (*n* = 3). Asterisks indicate values significantly different (*p*<0.05) from that of OATP1B1 wild-type.

### Uptake activity of mutants for higher concentration of estrone-3-sulfate

**Figure 6:**
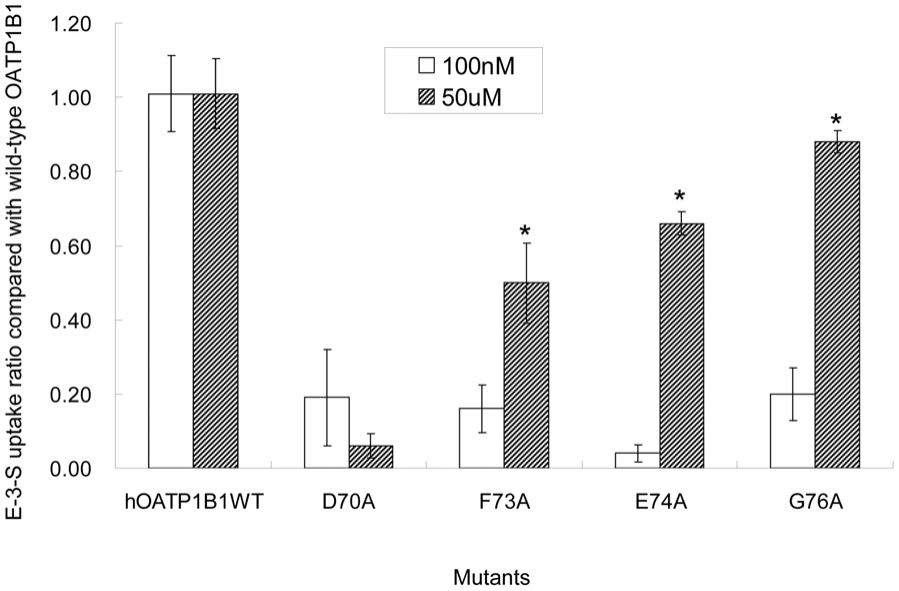
Effect of alanine substitution on uptake of high concentration of estrone-3-sulfate. Transport of 100 nM or 50 µM E-3-S in HEK293 cells expressing OATP1B1 or its alanine-substituted mutants was measured. Net uptake was obtained by subtracting the uptake of cells transfected with empty vector from cells expressing wild-type OATP1B1 or its mutants. Uptake of mutants was normalized to transporter expression at the cell surface in the same batch of cells and quantified relative to that of the wild-type. The results shown are means ± S.E. (*n* = 3). Asterisks indicate values that are significantly different (*p*<0.05).

**Figure 7:**
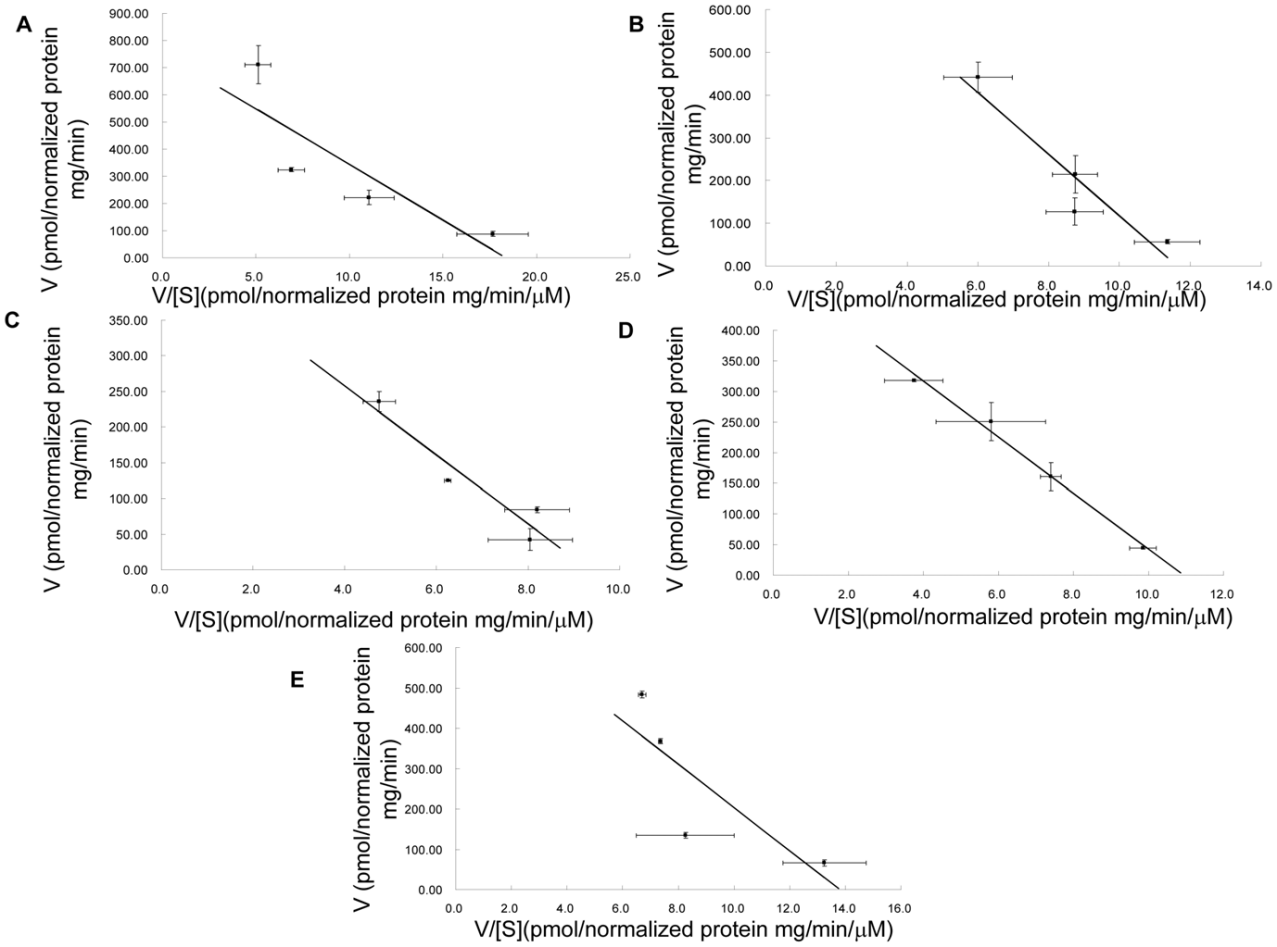
Eadie-Hofstee plots of high concentrations of estrone-3-sulfate uptake by OATP1B1 and its mutants. **A**. Uptake of OATP1B1, **B**.D70N, **C**. F73A, **D**. E74A and **E**.G76A. Uptake of E-3-S was measured at concentrations range from 5 to 100 µM. Uptake was conducted at 37°C for 1 min with empty vector, OATP1B1 or its mutants in HEK293 cells. Net uptake was obtained by subtracting the uptake of cells transfected with empty vector from cells expressing wild-type OATP1B1 or its mutants. Uptake of mutants was normalized to transporter expression at the cell surface in the same batch of cells and quantified relative to that of the wild-type. The results shown are means ± S.E. (*n* = 3).

It has been proposed that OATP1B1 has more than one binding site and that its transport of E-3-S displayed biphasic saturation kinetics with two distinct affinity components [10], [15], [20]. In order to see whether the substitution of these four amino acids also has effect on the transport function of higher concentrations of E-3-S, we measured uptake activity of the alanine mutants for 50 µM of E-3-S. As shown in Figure 6, net transport activity of all mutants had a significantly higher transport function except D70A. This implicated that Asp70 is involved in both low and high affinity interaction sites of E-3-S, while other amino acids identified may have more impact on the high affinity component of E-3-S. We further analyzed the kinetic parameters of these mutants for high concentrations (>5 µM) of E-3-S. As shown in Fig. 7 and Table 2, D70N, which was used to measure the kinetic parameters instead of D70A, of which uptake was too low for the analysis, demonstrated a significant increase of Km. For all the other mutants, Km and Vmax were comparable with those of the wild-type OATP1B1.

**Table 2:** Kinetic parameters of OATP1B1 wild-type and its mutants for high concentrations of E-3-S.

	Km (µM)	Vmax (pmol/mg protein/min)
OATP1B1	42.18±10.71	766.92±132.03
D70N	71.82*±4.76	785.59±205.18
F73A	44.35±7.54	424.17±49.33
E74A	51.04±17.42	531.68±117.54
G76A	51.68±1.34	721.94±36.73

Uptake of E-3-S was measure at concentrations of 5 µM, 20 µM, 50 µM and 100 µM at a 1-min interval for OATP1B1 and its mutants. Transport kinetic values were calculated using the Eadie-Hofstee transformation. The results shown are means ± S.E. (*n* = 3). Asterisks indicate values significantly different (*p*<0.05) from that of OATP1B1 wild-type.

## Discussion

Although the vital importance of OATPs in body drug disposition is indisputable [21], our knowledge on the structure-function relationship of OATPs is still limited. As a critical structural feature of membrane proteins, amino acids within TMs have been shown to play important roles in proper functions of these proteins [13]–[16], [19]. In the present study, we demonstrated the mutation of four amino acids (Asp70, Phe73, Glu74 and Gly76) within TM2 of OATP1B1 into alanine led to a significant reduction in the transport of E-3-S. Cell surface expression of these four mutants was also decreased compared with that of the wild-type (∼60%), suggesting that the substitution of these amino acids into alanine may partially affect the proper targeting of the transporter protein onto the membrane. However, the decline in transport function was much more dramatic and thus could not be totally accounted for by the reduction of cell surface protein level.

Both Asp70 and Glu74 are negatively charged amino acids. However, they seemed to affect transporter function in different ways. Although mutation of Asp70 into alanine significantly reduced transport activity (Fig. 1), substitution of the amino acid into glutamic acid or non-charged asparagine partially recovered the transport activity of E-3-S and also resulted in increased cell surface expression of the protein. Kinetic study on D70N showed an increased Km value as well as a decreased Vmax, suggesting Asp70 is involved in the interaction with substrate and that its side chain characteristics are crucial for the binding of E-3-S. On the other hand, when Glu74 was converted into asparatic acid, the mutant showed a significant uptake activity (∼50% of wild-type). Moreover, both Km and Vmax of E74D was similar with that of OATP1B1. That the charge characteristic of Glu74 is essential for OATP1B1 activity was further proved by the consequences of substituting this amino acid with glutamine, an amino acid that has similar structure but is non-charged. The substitution of Glu74 with glutamine still resulted in loss of transport activity (Fig. 3B), though cell surface expression level of E74Q was comparable with that of wild-type. Charged amino acids located within the transmembrane domain have been suggested to be critical for transport function, possibly through the formation of a salt bridge with a nearby oppositely charged amino acid [22]. Our results thus suggested that Glu74 may interact with positively charge amino acids located in the C-terminal half of the transporter protein and form a salt bridge to stabilize the binding pocket.

**Figure 8:**
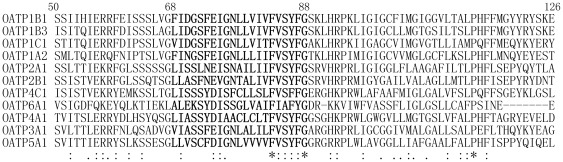
Comparison of TM2 sequences within OATP family. Full length sequences of 11 OATP family members were aligned with ClustalW. Only partial sequences were shown here. The corresponding sequences of TM2 were in bold.

**Figure 9:**
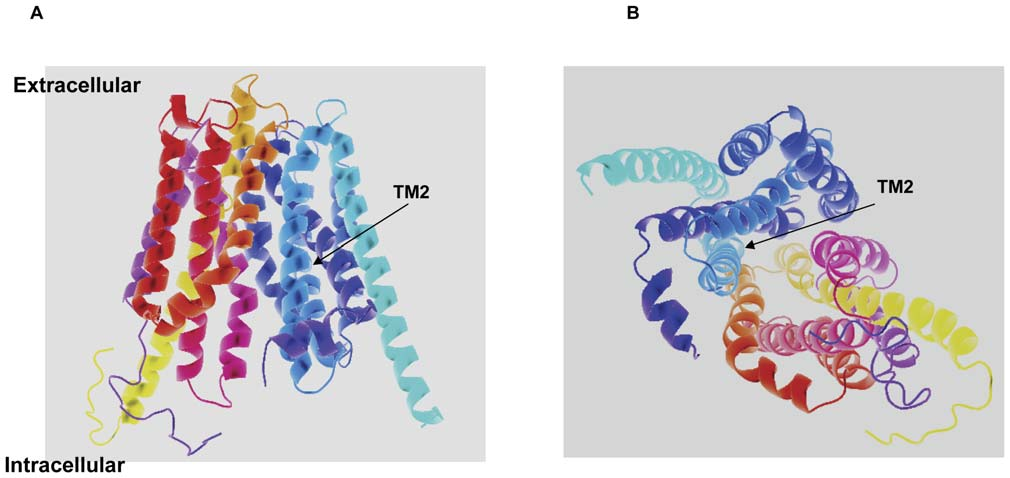
Putative computer model of OATP1B1. Viewed from **A.** the lateral side. **B.** the intracellular side. The glycerol-3-phosphate transporter (PDB 1pw4) was used as the template for homology modeling of OATP1B1. The structure of OATP1B1 was modeled with the web-based protein structure prediction service Phyre2 (http://www.imperial.ac.uk/phyre/). Arrows indicate that TM2 is located on the pore-facing side.

F73 is a relatively conserved amino acid within the OATP family. The line-up of all 11 human OATP members showed that 6 out of the 11 OATPs have a phenylalanine at the corresponding position, while 3 others have a tyrosine at this site (Fig. 8). We then postulated that the aromatic ring may be a critical factor in substrate binding and substituted Phe73 with tyrosine. The net transport function of F73Y with substrate E-3-S was around 40% of wild-type. Moreover, kinetic study showed that the K_m_ of F73Y is similar to that of the wild-type, which indicates the important role of the aromatic structure in its interaction with the substrate, possibly through a π-π interaction. However, the Vmax of F73Y was much lower than wild-type OATP1B1, suggesting a lower turnover rate for the mutant. Interestingly, F83 and F87 are more conserved than position 73, with all 11 or 10 out of 11 OATP members containing the same aromatic amino acid. However, substitution of these two positions with alanine did not seem to affect transport function of OATP1B1 significantly (Fig. 1). Gly76 is also a relatively conserved amino acid in the OATP family, with 7 out of 11 family members containing the same residue. We first postulated that Gly76 may be located within the transporting channel based on the homology modeling of OATP1B1 using glycerol-3-phosphate 1pw4 as the template (Fig. 9), of which Phe73 and Glu74 were implicated to face towards the pore. Considering the structure of an α-helix, in which amino acids spaced 3 and 4 apart in the sequence are spatially quite close to one another, Gly76 may be located in proximity to Phe73 and Glu74 and interact with the substrates. However, the substitution of glycine with valine, a residue with larger side chain than alanine, did not seem to result in more change of the transport activity. Conserved glycine residues are thought to result in breaks and bends in the TM helices [22]. The breaks and bends that are generated by glycine residues were suggested to cause the major part of the corresponding transmembrane helix to face the pore [23] and this is probably the case for Gly76. When it was mutated into alanine or valine, the curved nature of the helix changed and the part that is originally facing the pore may be turned away, thus resulting in the loss of transport activity.

OATP1B1 was proposed to contain more than one binding sites [20] and that its transport of E-3-S displayed biphasic saturation kinetics with two distinct affinity components [10], [15], [20]. To further analyze if the mutation of these four identified amino acids also has effect on uptake of high-concentration of E-3-S, we measured the transport activity of all four alanine mutants for 50 µM of E-3-S. Interestingly, except D70A, transport function of all the other mutants was significantly increased. This indicated that Phe73, Glu74 and Gly76 may be only critically involved in one binding site of OATP1B1, while Asp70 play a role in substrate binding of both sites. Our kinetic analysis seemed to support such a hypothesis because the Asp70 substitution mutant D70N showed a significantly higher Km value.

According to our computer model of OATP1B1 (Fig. 9), TM2 is facing the pore in which the interaction with substrates occurs. This is consistent with our mutagenesis study that identified four important amino acids in TM2 for the uptake of low concentrations of E-3-S. Asp70, Phe73, and Glu74 are probably localized on the pore-facing side of the transporter protein and involved in substrate binding or structure stabilizing. Gly76 on the other hand, may not directly interact with the substrate, but generates a break or bend that leads to the proper turning of the helix. That the uptake of higher concentrations of E-3-S was still lacking in mutant D70A suggested that this particular amino acid may be involved in both the low and high affinity component of E-3-S, while all the other three identified amino acids play roles in only one site. A recent study on conserved positively charged amino acids in OATP1B1 demonstrated that amino acids K90, H92 and R93 that are immediately adjacent to the TM2 had an impact on the functional properties of OATP1B1. Although these amino acids are located intracellularlly and away from the critical amino acids identified in our study, which localized on the extracellular half of TM2, their conservativeness and involvement of transport activity seem to support our findings that TM2 of OATP1B1 is essential for its transport function [24].
