# Correction: Identification of Amino Acids Essential for Estrone-3-Sulfate Transport within Transmembrane Domain 2 of Organic Anion Transporting Polypeptide 1B1

**DOI:** 10.1371/annotation/35943d4c-3455-4818-976a-be73a2e07fff

**Published:** 2013-05-09

**Authors:** Nan Li, Weifang Hong, Hong Huang, Hanping Lu, Guangyun Lin, Mei Hong

There was an error in the Funding statement. The correct Funding statement is available below:

This work was supported by Guangdong Province Talent Introduction Project Grant and National Natural Science Foundation of China grant (81141115) to MH. The funders had no role in study design, data collection and analysis, decision to publish, or preparation of the manuscript. 

